# Editorial: Clinical and Pathophysiological Peculiarities of Headache in Children and Adolescents

**DOI:** 10.3389/fneur.2019.01280

**Published:** 2019-12-06

**Authors:** Massimiliano Valeriani, Ishaq Abu-Arafeh, Aynur Özge

**Affiliations:** ^1^Headache Center, Ospedale Pediatrico Bambino Gesù, IRCCS, Rome, Italy; ^2^Center for Sensory-Motor Interaction, Aalborg University, Aalborg, Denmark; ^3^Paediatric Neurosciences Unit, Royal Hospital for Children, Glasgow, United Kingdom; ^4^Department of Neurology, Mersin University Medical Faculty, Mersin, Turkey

**Keywords:** headache, child, adolescent, developing brain, peculiarities

Headache is a very common disorder in adults as well as in children and adolescents. Tension-type headache, migraine, and medication overuse headache are the most prevalent neurological diseases (1) and, therefore, it is not surprising to see a large number of published studies on the clinical characteristics, pathophysiology, and treatment of headache disorders. Despite its high prevalence in children and adolescents and despite being the most common neurological condition for which children are referred for specialist pediatric neurology services, headache continues to be underdiagnosed and undertreated. There are no easy answers for the causes of underdiagnosis and undertreatment of childhood headache. However, it is possible that lack of attention and lack of recognition of the specific peculiarities of the different types of headaches in children may play a major part in this problem. Furthermore, and unfortunately, the international classification of headache disorders in their successive editions do not discuss the pediatric presentation of the different headache disorders except for certain aspects of migraine, giving the erroneous impression that primary and secondary headaches in children are just smaller versions of their counterparts in adult.

In assessing headaches in children, it is important to take into consideration the following major points: Firstly, the clinical, features, presentations, trigger factors, relieving factors, and interpretation of children's behavior during attacks of migraine and tension-type headaches, the most frequent types of primary headaches, can be very different, particularly in young children as compared to adults. Secondly, there is some evidence to suggest that genetic factors, pathophysiological mechanisms, brain development, and maturation of cerebral networks during childhood can influence the presentation of headache disorders in different age groups. Thirdly, children's responses to pharmacological and non-pharmacological treatment of migraine are shown to be different in the pediatric population. The placebo response has been shown to be so powerful in children to make it difficult to interpret the results of clinical trials of acute and preventive treatments (2). Fourthly, common secondary headaches in adults are much less common in children such as cerebrovascular diseases, substance misuse and psychiatric disorders, etc.

The present Research Topic aims to collect clinical observations and experimental evidence highlighting the peculiarities of headaches at this early stage of life. Eleven papers were published as part of the Research Topic and they all made useful contributions in showing how large and varied is the world of pediatric headaches. Most studies discussed issues related to diagnosis and two studies addressed treatment options.

Looking at the published studies in details, four papers (Moavero et al.; Papetti et al.; Toldo et al.; Parisi et al.) investigated the applicability of the 3rd edition of the International Classification of Headache Disorders (ICHD3). It was shown that, although the ICHD3 is a very useful tool for the diagnosis of headache even in pediatric age, some peculiar clinical characteristics, e.g., the shorter headache attack duration especially in pre-school children, have not been included. Moreover, Toldo et al. underlined the differences in the clinical presentation of hemiplegic migraine between children and adults. On the issue of ictal epileptic headache in children, Parisi et al. suggest that ICHD3 criteria are too vague and, in particular, fail to consider the diagnostic role of the immediate remission of EEG abnormalities and headache after intravenous administration of an anti-epileptic drug.

Cuvellier analyzed prodromic and postdromic symptoms in children and adolescents with migraine. He proposed the interesting hypothesis that these symptoms may shed light on pathophysiological mechanisms that may be specific of the developing brain.

Raucci et al. reviewed the literature concerning the management of headache in the emergency department. This is a clinically relevant issue as life-threatening etiologies of headache exist in any age including children, but fortunately less common than in adulthood. Since the misdiagnosis of these conditions can lead to serious consequences, clear red flags are necessary to improve the safety of young patients and to avoid inappropriate invasive examinations and investigations.

When dealing with pediatric migraine, comorbidities should always be considered. The study by Roccella et al. showed a reduction of arousability and lower NREM sleep instability associated with migraine without aura. This subject deserves further investigation in the future, since it could have important therapeutic implications.

In children and adolescents with migraine, psychiatric comorbidities can have a key role in determining the severity of the disease. Both studies included in the Research Topic (Genizi et al.; Sciruicchio et al.) suggest that personality traits, such as abnormal processing of sensory information and pain catastrophizing, modulate the clinical presentation of migraine and can be fundamental in its chronification.

Although new promising drugs for the treatment of migraine, such as the antiCGRP agents, have been introduced, their use will be limited to adulthood for the next years, since the clinical trials in children and adolescents have just started. The encouraging response to non-invasive stimulation of the brain in the treatment of headache in adults prompted its adoption in pediatric practice, as suggested by Brighina et al. The non-pharmacological interventions for migraine treatment in children, reviewed by Andrasik et al., bear an important role, also in consideration of the side effects associated with the use of preventive drugs, and the limited evidence about their efficacy (2).

In conclusion, we believe that the studies included in this Research Topic highlight eloquently the important differences between adult and childhood headache in its clinical characteristics, diagnostic criteria, pathophysiology and in the treatment approaches. These studies will, hopefully, help in establishing childhood headache as a special entity and not just a “small version of adult headache.” Investigating the pathophysiology and the clinical features of children's headaches is mandatory for primary headaches, whose genetic background can be unveiled, with a lower incidence of environmental factors than in adults. Also the treatment of pediatric headaches should not merely mirror that of adult headaches, since pharmacological and non-pharmacological therapies in children and adolescents should take account of the characteristics of the developing brain and the comorbidities typical of this age.

## Author Contributions

All authors listed have made a substantial, direct and intellectual contribution to the work, and approved it for publication.

### Conflict of Interest

The authors declare that the research was conducted in the absence of any commercial or financial relationships that could be construed as a potential conflict of interest.
